# Taking care of oneself by regaining control - a key to continue living four to five decades after a suicide attempt in severe depression

**DOI:** 10.1186/s12888-017-1223-4

**Published:** 2017-02-13

**Authors:** Lisa Crona, Margaretha Stenmarker, Agneta Öjehagen, Ulrika Hallberg, Louise Brådvik

**Affiliations:** 1 Department of Clinical Sciences, Psychiatry, Lund University, Lund, Sweden; 2 Futurum – Academy for Health and Care, Jönköping County Council, Jönköping, Sweden; 3 Former Nordic School of Public Health (NHV), Gothenburg, Sweden

**Keywords:** Severe depression, Suicide attempt, Professional care, Long-term course, Qualitative, Grounded theory

## Abstract

**Background:**

Depression is a strong risk factor for suicide and suicide attempt. Several studies have examined the pathway to suicide attempt, but few studies have considered aspects important for overcoming being suicidal. The aim of the present study was to examine personal strategies to continue living after a suicide attempt.

**Methods:**

A qualitative grounded theory approach was used. Thirteen former inpatients diagnosed with severe depression (1956–1969) participated in a follow-up 42–56 years after their last suicide attempt, which occurred between the ages of 21 and 45. They were interviewed on one occasion between June 2013 and January 2014, using semi-structured interviews.

**Results:**

The pathway to a suicide attempt was defined as ‘being trapped in an overwhelming situation’. Three categories described the recovery process: ‘coming under professional care’, ‘experiencing relief in the personal situation', and ‘making a decision to continue living’. These categories emerged in a core category, labelled ‘taking care of oneself by regaining control’. Overcoming being suicidal occurred regardless of recovering from depression.

**Conclusion:**

In the very long-term course following a suicide attempt, the process of recovery is multi-dimensional and fluctuating, and includes appropriate treatment, connecting with others, decision making, and overcoming existential issues.

## Background

Over 800 000 people die from suicide every year and many more attempt suicide [1]. Affective disorder is the single disease entity behind most suicides worldwide [2], and major depressive disorder with melancholic or psychotic features/endogenous depression has been shown to predominate among depressed suicide victims [3, 4]. As much as 15–34% of patients with major depressive disorder have attempted suicide [5, 6] and previous suicide attempt is a risk factor for accomplished suicide [7, 8]. It is therefore important to try to understand suicidality in this group.

Many studies have aimed to identify causes or risks of suicide and the underlying pathology or predisposing genetic, psychological or biological factors, such as mental health issues, comorbidity, ethnicity, family history, marital status, and occupation [9]. Important risk factors found are previous suicide attempt, male gender, psychiatric comorbidity, and highly lethal method regardless of intent [8, 10].

Despite the substantial literature on suicide attempt, Cutcliffe [11] points out that there are still many gaps in our knowledge and understanding of the experience and meaning of a suicide attempt. Qualitative research is a way to generate knowledge [12], by constantly asking questions about how people think, feel and act in different situations [13].

Several studies have considered the pathway to suicide attempt. Pavulans et al. [14] described the experience of being suicidal and making a suicide attempt in a core category ‘being in want of control’. Michel et al. [15] found that the main motive reported for attempting suicide was an acute and unbearable state of mind. Other studies concern aspects important for overcoming being suicidal [16–18]. Lakeman & Fitzgerald [16] reviewed qualitative articles on the topic and concluded that overcoming being suicidal involves various struggles, often existential in nature. People may turn away from suicidal behaviour quite abruptly by experiencing the right kind of connection with others. Two qualitative studies from Taiwan, with a follow-up time of around one year, focused on the healing process after a suicide attempt. One of the studies compared the process to an emotional navigation wheel, meaning that the process is not linear [17]. The other study underlined the role of “striving to accept the value of self-in-existence’ [18].

In a study of elderly suicide attempters, three broad themes emerged: struggle, control and visibility [19]. Bostik & Everall [20] investigated how adolescents overcame being suicidal; the process was associated with forming at least one close relationship and a growing sense of control that replaced perceptions of helplessness. In a study of young male suicide attempters, the nursing role was found essential, and the study highlights the importance of a supportive mental health professional who took a holistic perspective and met the patient with a supportive approach [21]. These results are in line with the findings of Cutcliffe et al. [22, 23]. The participants clearly felt that a close relationship with someone who could sit with them and discuss the near death experience was more important than being treated with drugs.

Personal contact therefore seems crucial in the acute phase after a suicide attempt, but little seems to be known about important factors in the subsequent very long-term perspective. The present study concerns former inpatients with severe depression admitted in 1956–1969 to the Department of Psychiatry, Lund University Hospital, who were followed up 42–56 years after their last suicide attempt.

### Aim

The aim of this study was to increase knowledge about important factors relating to the desire to continue living after a suicide attempt in persons with severe depression, in the very long-term perspective. A qualitative approach was applied.

## Methods

### Grounded theory

The method of modified grounded theory [24] was used for collecting and analysing data in the present study. Modified grounded theory is a developed form of classical grounded theory [25] but both versions are aimed at developing a theory or model that can explain and predict the phenomenon under study. Classical grounded theory tends to deal with how people handle different situations, and how they try to resolve the challenge [25], i.e. the main concern, for example when physicians are breaking bad news [26]. In modified grounded theory, the goal is to try to understand the personal meaning of a certain event and the social processes and behaviours related to the event [24], for example patient perceptions in medical care [27]. However, the basic principles of grounded theory always include constant comparisons, theoretical sampling, saturation and theoretical sensitivity. Constant comparisons include one piece of data being constantly compared with other pieces of data. Concepts must earn their way into the data [25] and different parts of the data are continuously compared in terms of differences and similarities. Also, a specific category is compared with other categories, as well as data from different subjects, and new data is compared with an emerging category. During the analysis, the developing category guided further data collection in terms of what to focus on in the interview. This approach led to changes in the interview items, and some questions were reformulated between interviews, i.e. theoretical sampling. The theoretical sampling procedure aims to refine theoretical ideas and saturating emerging categories. Saturation was reached when the research team jointly decided that all new data fitted into existing categories and that there was no theoretical basis for new categories [28]. Furthermore, the theoretical sampling was based on age distribution from the first and the last suicide attempt, and the participants represented several different professions.

Theoretical sensitivity refers to the researcher’s reflexive way of developing research questions and performing analysis [29]. In this study, the sensitivity was based on authors MS, UH and AÖ all having previous experience of the research method, author LB’s experience as a psychiatrist, and author LC’s experiences of earlier interviews. Our different professions and different experiences helped us try to control interview bias. This was a constant source of reflection during the research process. The dependability of this study relies on the fact that the same interviewer who conducted the original telephone interviews also conducted the interviews in this study.

### Study participants

#### Selection of the sample

**Fig. 1:**
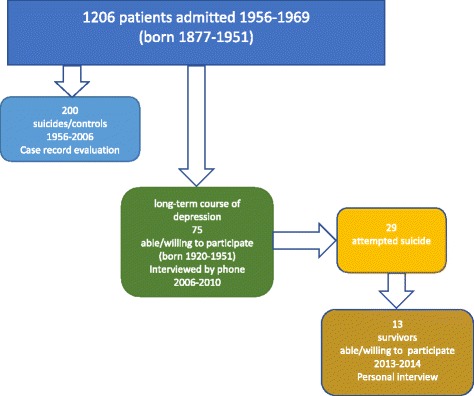
Flow-chart of persons who had been admitted to the Department of Psychiatry, Lund, University Hospital 1956–69 with a severe depression/melancholia. (Patients who had developed severe depression secondary to alcohol use disorders were excluded)

The study participants in the present study had all been admitted to the Department of Psychiatry, University Hospital, Lund, in 1956 up to 1969 and had received the diagnosis ‘severe depression/melancholia’ by a senior psychiatrist. Between 1949 and 1969 all in-patients were rated on a multidimensional diagnostic schedule on discharge [30], and from 1956 onwards, the diagnosis ‘severe depression/melancholia’ was used. This diagnosis was given to 1206 patients (700 women and 506 men born 1877–1951) up to 1969 (Fig. 1). Validation against DSM IV criteria has been performed in a case-record study on suicide victims (who died 1956–2006) and matched controls in this sample, in all 200 patients [31]. Good agreement was found, as 91% of these could be shown to meet the criteria for major depressive disorder with melancholic or psychotic feature, regardless of suicidal outcome.

In 2006, a survey of long-term depression was performed. There were 150 persons born from 1920 and onwards, who were alive and considered eligible. Out of those 75 were able/willing to participate in an interview by phone performed by LC [32]. This sample included 29 individuals who could be defined as “suicide attempters”. In 2013, 21 of them were still alive (one had committed suicide, one had died by undetermined intent, and six had died of other causes). They were contacted by phone by LC and asked to attend a qualitative interview in a personal meeting. Four could not be reached and four declined due to old age, sickness, or because they did not want to bring up their depression again. A total of 13 subjects agreed to participate and they were informed that they could withdraw at any time.

#### Characteristics of the sample

Demographic data are presented in Table 1. Life-charts including severity of depression and suicide attempts were presented in the previous study [32]. Remission, recovery, relapse and recurrence were defined as introduced by Frank et al. [33]. A cluster analysis was performed, and six course clusters were identified describing the course of depression [32]. In summary, there was a short-term course with or without recurrence, or a chronic course with or without late remission. Suicide attempts usually occurred early in course of depressive episodes [34]. In the present sample of previous suicide attempters, six persons had a short-term course without recurrence, three had a late recurrence, and four had a chronic course. Eight persons had at least one severe episode after the suicide attempt. The course of depressive episodes had lasted an average of 28 years (SD +/- 15), range 5–50 years (median 36).

**Table 1:**
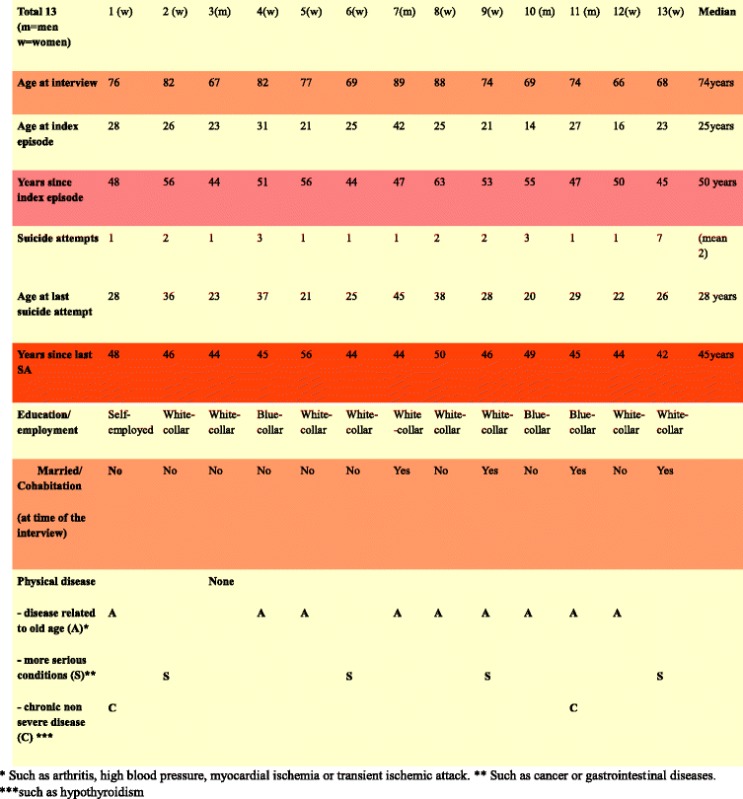
Demographic data, including age and time passed since index episode of depression and last suicide attempt

None of the subjects had dual mental diagnoses or alcohol problems, and one had misused tablets but had overcome the problem. Everyone in the study population had been treated with antidepressants and/or electroconvulsive therapy (ECT) and some had been given additional psychotherapy. All respondents had been working after their first depressive episode. One was not working later in life because of somatic illness. Three had ongoing depressive episodes throughout life and four had a relapse of depression at older age, but no new suicide attempts were reported. The median age at last suicide attempt was 28, range 21–45.

### Data analysis

The participants were interviewed on one occasion between June 2013 and January 2014 by LC. The interviews lasted between 45 and 110 min. The participants were able to choose the place of interview, either at their home or at the University Hospital in Lund, and gave their written consent. All the interviews were tape recorded with a portable microphone, except one that was recorded in writing instead, in accordance with the participant’s wish. The taping was of good quality.

The interviews were semi-structured using an interview guide with 23 questions arranged in four themes. The first theme was: life situation now and since last interview, for example “How are you feeling today? Has anything important happened since we last talked?” The second theme was: thoughts about causes of depression and factors of importance for recovery, for example “What do you think about your depression, now and then? What was important that became tough in your life, and what made you feel better/well?” Third: thoughts about the suicide attempt, causes and factors important in the decision to continue living after the attempt, for example “You once attempted suicide, what made you do that? How was your life situation at that time? Your thoughts and feelings? What made you choose life again? How was that process?” Finally, the fourth theme was: relationship between depression and suicide attempt, for example “What role did the depression play? Other things that happened to you? Were the depression and the suicidal thoughts connected? Or did one thing end before the other?”

The inductive questions were based on a literature review and earlier telephone interviews. The deductive questions in the interview guide were then developed using open-ended answers. The first question was an introduction to the interview and not analysed further. In line with grounded theory methodology, the interview guide was modified during the research process; some questions were reformulated and some questions were added. The interviews were transcribed literally, so every word expressed in the interview was written down, throughout the research process.

The text was analysed in an open, axial and selective coding process [24, 35] by authors LC, MS and UH. The aim of the analysis was to provide an explanatory process describing the journey from a suicide attempt to continue living many years after. We also identified patterns and relationships within data that seemed crucial for this process.

- Open coding: after each interview had been transcribed, the data was read line by line to capture the substance and coded into significant statements that formed emerging concepts. Concepts with similar meanings were grouped together in tentative categories (*n* = 18) using the constant comparative method. The categories were either labelled with actual words used by the participants (in vivo codes) or labelled with words from the professionals in the research group (in vitro codes).
- Axial coding: Each category was further developed in terms of properties and dimensions, and relationships between categories were identified according to the constant comparative method. Four major categories were defined. These categories were given more abstract names than the codes belonging to them. The categories were saturated by assessing new interviews or by recoding previously assessed data.
- Selective coding: A core category was identified that was central in the data and that all other categories could be related to and integrated with. This led to the description of a psychosocial process symbolised with a figure.

Throughout the analysis process, ideas, preliminary assumptions and theoretical reflections were recorded in writing in memos [24].

## Results

**Fig. 2:**
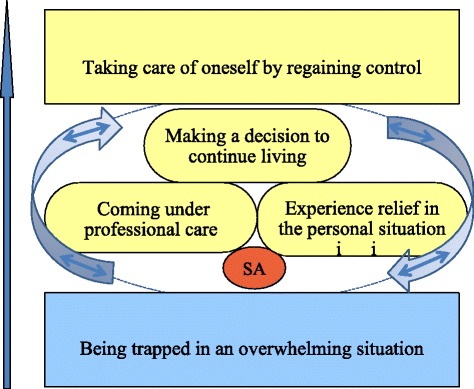
The way to regain control is characterised by a process that fluctuates between the different steps illustrated by the labelled categories

A core category for strategies after the suicide attempt was found and labelled “taking care of oneself by regaining control”. Four categories were identified. “Being trapped in an overwhelming situation” describes the pathway to the suicide attempt. The three other categories concerned the period after the suicide attempt: “coming under professional care, experiencing relief in the personal situation, and making a decision to continue living”. The way to regain control is characterised by a process that fluctuates between the different steps, and the study participants emphasise different parts of the process (Fig. 2). In each category illustrative quotations are presented in italics.

### Being trapped in an overwhelming situation

The respondents described feeling that they were trapped in overwhelming situations before the suicide attempt. Excessive demands were often experienced simultaneously in the private, social and professional arenas. For example, it could be a problematic relationship with their partner, parallel with an excessive workload and having a young family. This was often combined with the respondent experiencing a feeling of vulnerability and a feeling of physical and psychological loneliness, which could have its origin in a problematic childhood or adolescence, or just a general feeling of weakness in the personality. For example one participant said: “If you’re alone everything gets a little worse.” For some participants, depression or other psychiatric illnesses were present in the family. The symptoms of depression could develop slowly step-by-step and the respondent gradually became aware of them over a period of time, as another one stated:“It was an endogenous thing; it was my body, total dissolution”, or the depression could start suddenly and take the respondent by surprise. One participant expressed: “It was like everything disappeared, everything became meaningless, a complete reduction of light… I lost my foothold.”

In both cases the depression led to thoughts of death and dying, with the participants thinking of death as a solution to their problems. The respondents described how they either carefully planned their suicide attempt, such as by storing medication for a long time, or tried to commit suicide in the heat of the moment, such as by taking too many tablets spontaneously or even by shooting themselves. They described the feeling as something dark suddenly coming over them, everything becoming meaningless, and also expressed the situation as being no longer capable of resisting all the accumulated misery in their body, for instance someone said:“…my life became meaningless, I couldn’t see it in plain language, it was like I had been crawling down in a black garbage bag and someone closing it at the top.” The respondents either called for help themselves after the suicide attempt or were found by somebody else.

### Coming under professional care

After the suicide attempt the respondents came under professional care in various psychiatric wards. One participant said: “There was a change while I was at the hospital; I started to see life in brighter colour.”

During their stay at the psychiatric ward they received different kinds of treatments, such as antidepressant medication, ECT and psychotherapy. They felt that the treatment made their depression start to fade away or at least made it easier to handle and also contributed to the relief from suicidal feelings, as one of them expressed: “The therapy helped me structure my confused brain.” They could also feel that there was someone in the staff who cared for them a little bit more, such as the doctor or the psychologist. One interviewee said:“It was people, doctors and the medication that made me feel better.” The important thing was not who it was, but rather that it was somebody who they felt cared for them on a deeper and genuine level. The respondents described how this person became very important, and they remembered the person vividly even a long time after their encounter. The human encounter and the treatment were essential. One participant described: “When I was admitted to the hospital, I was in a ward with loads of people and it stopped, it became completely different somehow. Life became completely different, a change with people around you. The medication calmed the body down to another level.”

### Experiencing relief in the personal situation

The respondents described how they eventually experienced relief in their strained life situation. This relief could occur in combination with their stay in the psychiatric ward or later, quite long after their stay. It could be a reduced or changed workload in their professional life, a divorce from a partner in a problematic relationship, or some other kind of private, social or professional relief. Some found relief in music, nature or physical activity, for instance one person said:“Then I started to study, found new friends and started to get interested in politics.”

The relief could also consist of support from a close friend, as one of them stated: “… I had some very good friends who were very supportive during the whole period. It should not be forgotten either, that all the time I was at my worst, they kept contact. They came to visit me and they made an effort.” It could also be a partner, spouse or relative, someone the respondents trusted, felt confidence in, and who made them feel accepted. For example one participant said: “My brother says I should thank my wife that I am alive, which is true.” For some participants, relief came by giving support to others instead of receiving support. The support gave them a reasonable chance to be able to cope with their strained situation, and they could feel more in control, according to one of them: “I had a wonderful job; it helped me out of this depression.” However, this did not necessarily mean that the depression or symptoms of depression ceased – the respondents could still feel some kind of depressive symptoms and could also consider another suicide attempt.

### Making a decision to continue living

The respondents described how they then made a decision to continue living, at least for the foreseeable future, for instance “… and then, the day came when I made the decision myself.” They had made a conscious decision not to try to make another suicide attempt. This decision was not necessarily something they verbalised to their close relatives; it was rather a decision made privately, for themselves. According to one participant:“It was life that took a grip on me, I dared, I decided that I wanted to live as normal a life as possible.” They were sometimes aware that suicide could be a solution on another occasion, but not in the present situation. One interviewee said: “I thought, I won’t give up, I give myself five years and during that time I am going to fight. I can’t think about suicide one single time, I just can’t. I try and I fight.” Some could make a definite decision never to try to commit suicide again, irrespective of the circumstances, a decision that became a point of no return. For others it was a decision that grew over time, and a continued fight to survive. “I feel well, but I have to rely on these emotions and control my emotions in this direction to be able to feel well.” The decision was made regardless of the depression or the depressive symptoms, and thus not related to depressive symptoms. Some respondents made this decision when still feeling depressed while some made it during remission or recovery.

### Taking care of oneself by regaining control

The core category describes how the respondents finally regained control of their situation and also started to take care of themselves. For example one participant said: “I realised that I was the only one who could control my own life, no one else.” The regained control was a result of both external circumstances like medication, support from others, or a change in the strained situation, and internal circumstances like the decision to continue living. This process led to a feeling of change in themselves as persons, becoming either more humble or tougher. One of them stated: “You become stronger by taking care of yourself.” They felt that their experiences had changed them on a deeper level and had also improved their ability to take care of themselves. The respondents could, for example, avoid excessive pressure and an excessive workload, or avoid certain people, in order to improve the way they felt. For others, more responsibility at work or in private life, or gaining a purpose in life, was a way of regaining control. This regaining of control and the urge to take better care of themselves was regardless of whether or not the depression was still present. According to one participant: “The suicide thoughts ended before the depression had disappeared.” Some of the respondents still felt depressed, at least occasionally, while others felt no depressive symptoms at all. For some people, this regaining of control could be a process with active awareness, while for others it was more of a passive conforming to life, as one of them expressed: “It was a slow upward curve, because when it started to turn, not suddenly but gradually, the living spirits came alive.”

## Discussion

This study presents the results of thirteen interviews conducted with former inpatients diagnosed with severe depression (1956–1969), in a follow up 44–56 years after the index episode of depression, and 42 to 56 years after their last suicide attempt. One category, “being trapped in an overwhelming situation”, described the pathway to the suicide attempt, and then a process of finding the way out was identified. This process comprised “coming under professional care and experiencing relief in the personal situation”, including better interrelations with others, then “making a decision to continue living”. The core category was labelled “taking care of oneself by regaining control”. The way to regain control is characterised by a process that fluctuates between the different steps, and the study participants emphasise different parts of the process.

The core category describes how the respondents started to take care of themselves and eventually regained control of their situation. This was as a result of a decision to continue living and feeling a gradual change over time, after undergoing many phases, as an individual. In a one-year follow-up, Chi et al. [17] proposed an emotional navigation wheel for healing and recovery following a suicide attempt in 14 subjects with depression. The authors described five phases: self-awareness, inter-relatedness of life, cyclical nature of human emotions, adjustment, and acceptance. While each phase might follow the preceding phase, it was not a linear process, and patients might move backwards and forwards between the phases. In the present study the process of recovery was multidimensional and fluctuated backwards and forwards in the same way as described by Chi et al. The very long-term follow up in this study reveals that such fluctuations may move on for several years and may even be life-long.

“Being trapped in an overwhelming situation” describes the pathway to depression and suicide attempt. Similar results were found by Pavulans et al. [14] and may represent the “cry of pain theory” proposed by Williams & Pollock [36]. Depression and thoughts of suicide then developed, either over time or were described as something dark suddenly coming over. The latter may be related to melancholia, and represents a more endogenous suicidality with overwhelming suicidal thoughts or unwanted suicidal urges, as described by Akiskal and Ringel [37, 38].

On the way to taking care of oneself by regaining control, it seems important to “come under professional care” and to experience “relief in the personal situation”, which concords with the conclusion from Lakeman & Fitzgerald [16] that people may turn away from suicide quite abruptly through experiencing, gaining or regaining the right kind of connection with others. In a grounded theory study by Cutcliffe et al. [22], the core variable was labelled ‘re-connecting the person with humanity’. In what the author calls a parsimonious theory, a three-stage healing process is described, consisting of the sub-core variables ‘reflecting an image of humanity’, ‘guiding the individual back to humanity’ and ‘learning to live’. Lakeman & Fitzgerald [16] express these results as regaining control over thoughts and feelings rather than being controlled by them. A connection with others was in essence what was perceived as therapeutic about psychiatric care and psychotherapy in one study [39].

Our study also highlights the importance of being a part of a supportive environment and having a feeling of being needed, in line with previous research [18, 40]. The present study indicates that a connection with others is a key factor, early in the healing process. As concluded by other authors [21–23], our results emphasise the importance of professionals in a supportive function. For some participants, this support was considered important for a long period and helped them to avoid relapse.

We did not find that the participants explicitly preferred to form a close relationship with a nurse rather than being treated with medicines, as proposed by Cutcliff [22, 23], but they emphasized the importance of finding someone who cared for them on a deeper level. The participants in the present study had all suffered from severe depression, and the role of pharmaceutical treatment and ECT was rated as being useful by the former patients, and probably very essential for persons with major depressive disorders being suicidal.

The subjects in our study also described how they “made a decision to continue living” on their way to taking care of themselves by regaining control. Self-reflection after many years perhaps lay behind a decision to continue living, a decision made privately without communication with others. It is notable that the decision was made regardless of the depression and the depressive symptoms, so some respondents could make this decision while still feeling depressed while others were recovered. Mann et al. [41] proposed a stress-diathesis model, where suicidal acts are not determined solely by psychiatric illness (the stressor) but also by a diathesis, such as a tendency to experience more suicidal ideations and to act more on suicidal feelings. Such a model corresponds with the present findings.

Finally, the core category was “taking care of oneself by regaining control”. The role of control has also been described in earlier studies, both the “lack of control” [42] and “being in want of control” as a general feature of being suicidal [14].

Consequently, regaining control appears to be a way to overcome feeling suicidal and to promote a desire to continue living, as has been found in the long-term perspective in our study. To the best of our knowledge, the category “taking care of oneself “has not been found in other studies. This theme seems to be crucial and may have emerged as a result of this very long-term study, and emphasising that it takes a long time to realise the effect of becoming more capable of taking care of oneself. Jordan et al. [20] further stress the importance of the treatment being future oriented. In the present study two steps in the process appear to be related to the long-term course. Firstly, in particular, the decision is taken alone, compatible with an existential point of view with an individual position taken to go on living. This existential view may be expressed by the words of Yalom [43]: “No matter how close each of us becomes to another, there remains a final unbridgeable gap; each of us enters existence alone and must depart from it alone.”. Secondly, staying alive and reproducing are the biological bases in attachment theory and “protection of self” a cornerstone [44]. These two steps are also in line with a more optimistic attitude towards life and may be an essential part of the individual's confidence in “being able to take care of oneself”.

### Strengths and limitations

The major strength of the study is the very long-term follow-up. We contacted all presumptive participants (*N* = 21), and 13 agreed to participate. Furthermore, the theoretical sampling, which is a cornerstone in grounded theory [23], was also based on age distribution from the first and the last suicide attempt and the participants represented several different professions.

The sample comprised former inpatients, which may not be representative for outpatients. However, suicide attempt in severe depression has been proposed to be an indication for admittance to hospital, which reduces the bias [45]. Participants were admitted to the psychiatric department at the hospital, and there was a mental hospital in the same area. This may skew the sample towards more middle-class patients or exclude those with very severe illness. However, several of the patients were also admitted to the mental hospital during more severe stages of illness.

It could be argued that the sample was skewed on account of the dominance of female participants (two out of three) and the age distribution. More men than women commit suicide but the opposite applies for suicide attempts [46], though in severe depression there seems to be similar rates of suicide and no clear gender difference in suicide attempts [31, 47]. The suicide attempts were made from adolescence to early middle age, and so may not be representative for suicide attempts in old age. Different factors have been found to be crucial for overcoming being suicidal in young [20, 21] and old age [19].

The rates of suicide attempts in the sample followed up was 39% (29/75), which is slightly higher than 34% for a subsample of controls to suicides [31], and 25% among controls at index admission [48]. This small discrepancy could be expected by a longer follow-up for each of the studies, so the present sample could be assumed to be representative of the total sample.

Connected with the great strength of a long-term follow-up comes the limitation caused by recall bias. However, the subjects in this study seemed to have a good memory of the suicide attempt. The first phases in recovery may be partly forgotten but, on the other hand, the long-term strategies could only be described later. The decision to continue living was sometimes taken during a depressive episode. However, it was difficult to get information of the severity of the depressive episode during which the decision was taken. Thus, we know that full recovery was not required but not if partial remission was.

However, it is important to note that the experiences of these former patients may differ from other patients’ experiences. When helping people in the recovery process after a suicide attempt, individualised treatment is always needed in order to provide the best help and care.

### Ethical discussion

The vulnerability in this group needs further ethical discussion. No one requested the follow- up support meeting offered after the interview, which had been offered in case anyone felt ill at ease after the interview. We can assume that nobody required such support. Some of the participants stated that it was, for them, a privilege to be interviewed. They were old and often lacked someone to talk to, and that someone listened to their stories seemed positive for most of them. There is something positive in reflecting over life even if it includes difficult periods and acts and, as highlighted by Lakeman et al. [49], there can be a psychotherapeutic benefit in participating in a research study.

## Conclusion

“Taking care of oneself by regaining control” appeared to be the main factor involved in overcoming the suicidal feeling over many years. In the very long-term course following a suicide attempt, the process of recovery is multi-dimensional and fluctuating, includes adequate treatment, connection with others, decision making and sorting out existential issues. Adequate somatic therapy (ECT and antidepressant pharmacotherapy) seems important, which should be adapted to the fact that the persons suffer from severe depression with melancholic and/or psychotic features. However, overcoming a suicidal feeling does not appear to be simply a matter of overcoming depression, and overcoming depression does not seem to be a prerequisite to overcoming being suicidal. Psychosocial interventions seem crucial, initially by support from at least one trusted person. In the long term, the difficulty seems to be more existential in nature, and the person makes a private decision to continue living and take care of themselves. It is crucial both to be supportive in the acute phase and more open to discussion about existential issues in other phases.
